# The Impact of Antenatal Azithromycin and Monthly Sulfadoxine-Pyrimethamine on Maternal Malaria during Pregnancy and Fetal Growth: A Randomized Controlled Trial

**DOI:** 10.4269/ajtmh.22-0496

**Published:** 2023-02-13

**Authors:** Lotta Hallamaa, Per Ashorn, Yin Bun Cheung, Mari Luntamo, Ulla Ashorn, Teija Kulmala, Kenneth Maleta, Charles Mangani, Yue-Mei Fan

**Affiliations:** ^1^ Center for Child, Adolescent and Maternal Health Research, Faculty of Medicine and Health Technology, Tampere University, Tampere, Finland;; ^2^ Department of Pediatrics, Tampere University Hospital, Tampere, Finland;; ^3^ Centre for Quantitative Medicine, Duke-NUS Medical School, Singapore;; ^4^ Department of Public Health, School of Global and Public Health, Kamuzu University of Health Sciences, Blantyre, Malawi

## Abstract

Maternal malaria and infections during pregnancy are risk factors for fetal growth restriction. We assessed the impact of preventive treatment in pregnancy on maternal malaria and fetal growth. Between 2003 and 2006, we enrolled 1,320 pregnant Malawian women, 14–26 gestation weeks, in a randomized trial and treated them with two doses of sulfadoxine-pyrimethamine (SP, control) at enrollment and between 28–34 gestation weeks; with monthly SP from enrollment until 37 gestation weeks; or with monthly SP and azithromycin twice, at enrollment and between 28 and 34 gestation weeks (AZI-SP). Participants were seen at 4-week intervals until 36 completed gestation weeks and weekly thereafter. At each visit, we collected dried blood spots for real-time polymerase chain reaction diagnosing of malaria parasitemia and, in a random subgroup of 341 women, we measured fetal biparietal diameter and femur length with ultrasound. For the monthly SP versus the control group, the odds ratios (OR) (95% CI) of malaria parasitemia during the second, third, and both trimesters combined were 0.79 (0.46–1.37), 0.58 (0.37–0.92), and 0.64 (0.42–0.98), respectively. The corresponding ORs for the AZI-SP versus control group were 0.47 (0.26–0.84), 0.51 (0.32–0.81), and 0.50 (0.32–0.76), respectively. Differences between the AZI-SP and the monthly SP groups were not statistically significant. The interventions did not affect fetal biparietal diameter and femur length growth velocity. The results suggest that preventive maternal treatment with monthly SP reduced malaria parasitemia during pregnancy in Malawi and that the addition of azithromycin did not provide much additional antimalarial effect.

## INTRODUCTION

Low birth weight (LBW) is an important risk factor of childhood mortality, morbidity, and lower intellectual ability later in life.^1^^–^^3^ The condition can result from preterm birth or fetal growth restriction, and it is most common in sub-Saharan Africa and Southern Asia. Despite being listed as a WHO nutrition target in 2012, the incidence of LBW has not decreased in recent years, and, according to recent estimates, more than 20 million infants are born with LBW each year.^4^

Typical interventions aimed to reduce LBW incidence focus on improving maternal nutrition, managing maternal infections, encouraging a healthy life style, and providing health services to pregnant women.^5^^–^^9^ In malaria-endemic areas, one of the most effective interventions is intermittent preventive treatment of malaria in pregnancy (IPTp).^10^ Results from a meta-analysis suggest that, in malaria-endemic areas in Africa, LBW prevalence can be reduced by approximately 20% with the provision of three or more doses of sulfadoxine-pyrimethamine (SP) to pregnant women, as compared with only two doses.^11^ Results from our own trial in rural Malawi suggested that the addition of azithromycin antibiotic to a monthly IPTp regimen (AZI-SP) would further reduce LBW prevalence and increase the mean weight, length, and head circumference of the infants at 1 month of age.^12^^,^^13^ Provision of AZI-SP to pregnant women was also associated with a lower incidence of stunting, better cognitive development, and possibly lower postneonatal infant mortality in their offspring.^14^ Thus, addition of azithromycin to the standard IPTp appears a promising intervention to improve birth outcomes and child health in some contexts. There is also evidence that periodic mass drug administration (MDA) of azithromycin to 1- to 59-month-old children reduces their mortality, and the WHO has issued a statement that such MDA may be considered in areas with high childhood mortality.^15^^,^^16^ However, it would be useful to know more about the possible mechanisms that mediate the positive effects of azithromycin on apparently healthy individuals.

In the current study, we aimed to determine the relative impact of the two interventions, compared with a two-dose SP IPTp regimen, on maternal malaria parasitemia in the second and third trimesters of pregnancy. Additionally, we assessed the impact of the two IPTp interventions on fetal growth velocity. We have earlier shown that monthly SP regimen with and without azithromycin reduced the prevalence of microscopy-diagnosed malaria at 32 weeks of gestation and polymerase chain reaction (PCR)-diagnosed malaria at delivery, without major differences between monthly SP and AZI-SP groups.^12^^,^^17^ Hence, we hypothesized that both interventions would reduce maternal malaria parasitemia prevalence throughout pregnancy in a similar manner. In contrast, we expected only AZI-SP to increase fetal biparietal diameter and length gain velocity due to earlier results in which infant length and head circumference at 1 month of age were greater in the AZI-SP group, but not in the monthly SP group, compared with the control group^13^ and because of the antibacterial and anti-inflammatory properties of azithromycin and the suggested negative influence of maternal infection and inflammation on fetal growth.^18^

## METHODS

### Background/study design.

This study is a secondary analysis of maternal PCR-diagnosed malaria parasitemia throughout pregnancy and ultrasound-assessed fetal growth as part of Lungwena Antenatal Intervention Study. The trial was a single-center, randomized, partially placebo-controlled, outcome assessor–blinded, three-arm clinical trial in rural Malawi. The original study hypothesis was that preterm delivery (i.e., the primary outcome of the study) and other adverse pregnancy outcomes could be reduced by IPTp with monthly SP alone or in combination with two doses of azithromycin.^12^^,^^17^

### Participants and follow-up.

Details of the inclusion and exclusion criteria and randomization are available in the original trial publication.^12^ In brief, we randomly allocated 1,320 women with ultrasound-confirmed gestational age of 14–26 weeks to either a control group or to one of two intervention groups: monthly SP or AZI-SP. Women in the control group received standard Malawian antenatal care, which at the time of the study included IPTp with SP (three tablets orally, each containing 500 mg of sulfadoxine and 25 mg of pyrimethamine) twice: at enrollment and at 28–34 weeks of gestation. At these visits, they also received a placebo in lieu of azithromycin. The second group of women received SP monthly from enrollment until 37 gestational weeks and two doses of placebo in lieu of azithromycin (monthly SP group). Women in the AZI-SP group received monthly SP and active azithromycin (two tablets orally, each containing 500 mg of azithromycin) twice: at enrollment and at 28–34 weeks of gestation. Both HIV-negative and HIV-positive mothers were enrolled in the study. HIV-positive mothers received nevirapine for prevention of mother-to-child transmission (PMTCT) during post-test counseling and were advised to take it at the onset of delivery, and HIV-exposed newborns received nevirapine syrup during the home visit after birth, which was the recommended care at the time of the study. The HIV-positive women did not receive co-trimoxazole, which became a national policy only after the study was conducted. Only participants who signed or thumb-printed an informed consent form were enrolled in the study.

All participants were seen at the health center at 4-week intervals until 36 completed gestation weeks and weekly thereafter. At each visit, the mothers underwent an interview, a routine antenatal investigation, and blood sampling. From the blood samples taken at the clinic, 100 μL (two spots, each 50 μL) was applied to Whatman FTA filter paper (Whatman plc, Maidstone, UK), air-dried, and placed in individually sealed plastic bags with a desiccant. The sample bags were stored in dry conditions at room temperature prior to transport to the Tampere University, Finland. The gathered dried blood spot (DBS) samples were analyzed by first extracting the DNA^19^ and then analyzing the sample with real-time PCR to detect the lactate dehydrogenase gene of *Plasmodium falciparum*.^20^ The percentage of infections with non-*falciparum* species was low among those who delivered and were tested at a health facility (0.6% for *P. malariae* and 0% for *P. vivax* and *P. ovale* with microscopy detection at delivery).^20^

Pelvic ultrasound to assess fetal biparietal diameter and femur length was performed for all study participants at the first antenatal visit and for a random subgroup of 341 women at each subsequent visit. Pelvic ultrasound was done with a portable analyzer by a research nurse (Aloka SSD-500; Aloka Co. Ltd., Tokyo, Japan, or Hitachi EUB 310; Hitachi, Ltd., Tokyo, Japan). Hadlock tables were used to calculate fetal age at the first visit.

### Outcomes.

To identify maternal malaria parasitemia status, all DBS samples were run on an ABI 7900 Real-Time System (Applied Biosystems, Foster City, CA). Samples were considered positive if both cycle threshold values were below 45. We aimed at high sensitivity and specificity and therefore analyzed all samples in duplicate. Reactions with only one amplification curve reaching the threshold line were repeated. Analyses of PCR-diagnosed malaria parasitemia included participants who had at least one DBS sample collected after enrollment and who had all the background information available that we used as covariates in the analyses. We have previously reported the prevalence of maternal PCR-diagnosed peripheral blood malaria parasitemia at delivery, but we excluded these samples from our main analyses because the samples had been analyzed with a different laboratory protocol.^17^

Biparietal diameter and femur length were measured in duplicate as millimeters. An average of the two measurements was used and rounded down. We excluded twin pregnancies because we could not confirm which fetus was measured. Of the 341 participants with repeated biparietal diameter and femur length measurements, the analysis included the results of 337 participants who had all background information available that was used as covariates in the analyses. We graphically compared the biparietal diameter and femur length in our sample to the 5th, 50th, and 95th percentile in the INTERGROWTH-21st standards.^21^

### Statistical analysis.

The sample size of 440 pregnant women per group was planned to give 80% power at a 5% level of significance to detect a 40% reduction in the rate of preterm delivery, which was the trial’s main hypothesis.^12^

Treatment allocation for the study was broken for the analysis of the trial’s main hypothesis. The statistician for this secondary analysis was different from the one doing the analyses for the main hypothesis. For this analysis the statistician (L.H.) merged the treatment allocation codes with maternal malaria parasitemia and fetal ultrasound data only after data were cleaned, the analysis plan was written, and the syntax for the analysis was done with a mock code. The analysis was based on the principle of intention-to-treat. We conducted statistical analyses with Stata 16.1 (StataCorp, College Station, TX).

For maternal malaria parasitemia during pregnancy, we calculated the proportion of positive malaria parasitemia DBS samples by intervention group during the entire follow-up period and for the second trimester (from 14 weeks + 0 days to 26 weeks + 6 days of gestation) and third trimester (from 27 weeks + 0 days of gestation to the end) of pregnancy. We estimated the odds ratios (ORs) between the three groups with mixed-effects logistic regressions with random intercepts by participant. Because of different timing of enrollment in terms of gestational weeks, the timing and number of the 4-weekly assessments per pregnancy varied. There were also missed visits during the follow-up. We used a mixed effects model that allows uneven timing and in which the random effect captures individual intercepts despite the variation in number of observations per participant. We estimated two different models: one for the entire follow-up period and one estimating the OR for the second and third trimesters separately. The model estimating the OR for the second and third trimesters separately allowed the estimates of treatment effect to differ between trimesters. Based on the OR, we estimated marginal risk ratios between the intervention groups from the model for the entire follow-up period.

We modeled biparietal diameter (mm) and femur length (mm) in relation to gestational weeks and intervention groups during the second and third trimesters of pregnancy by mixed-effects linear regression. Because fetal growth was not linear throughout pregnancy, we used two separate models to estimate the slopes for the second and third trimesters. The duration of pregnancy was centered to 14 weeks, which was the shortest duration of pregnancy at enrollment in the data. The model included random intercepts and random slopes by participants and allowed variation in the timing and number of ultrasound measurements per pregnancy. The random effects were allowed to be correlated. We estimated the change in biparietal diameter and femur length in relation to gestational weeks, with an interaction term between intervention groups and weeks of gestation, and the slope was multiplied by 4 to obtain the change per 4 gestational weeks. We calculated the differences in the rates of change between the three groups.

The proportion of women with none or one previous pregnancy and with microscopic peripheral blood malaria parasitemia at enrollment was higher in the control group than in the intervention groups.^12^ For analysis of maternal PCR-diagnosed malaria parasitemia, we included maternal PCR-diagnosed malaria parasitemia at enrollment, number of previous pregnancies, and duration of pregnancy at each visit in the models as covariates to account for the differences between groups in baseline characteristics and for the differences in the timing of antenatal visits during follow-up. For the analysis of fetal growth rate, we included duration of pregnancy, PCR-diagnosed malaria parasitemia at enrollment, maternal body mass index at enrollment, number of previous pregnancies, and the biparietal diameter or femur length measurement taken at enrollment as covariates. Because we analyzed fetal growth velocity with separate models for the second and third trimesters, we decided to use the enrollment measurements as a covariate to be able to include them in the third trimester analyses.

The null hypothesis of no difference between groups was rejected if *P* < 0.05. Wald’s test was used to test the global null hypothesis of no differences between groups and the pairwise comparisons. For pairwise comparisons with *P* < 0.05, the hypothesis of no difference between groups was rejected only if the global null hypothesis was also rejected.^22^

As an exploratory analysis, we performed tests for interaction between interventions and number of previous pregnancies (as a continuous variable), maternal HIV status, and bed net use at enrollment by using the likelihood ratio test and considered *P* < 0.1 as evidence of interaction. We performed all analyses stratified by the same variables. The same set of variables was used for stratification with the trial’s main outcomes.^12^ As a sensitivity analysis, we included maternal PCR-diagnosed malaria parasitemia at delivery to our outcomes for those participants who had it available, and we also estimated the rate of change in biparietal diameter and femur length without measurements taken at or after 38 weeks of gestation, a time point after which the observed growth velocity started to level off, making linearity assumption during the third trimester slightly less valid.

### Ethics committee approval.

Both the original trial and the follow-up were performed according to Good Clinical Practice and the ethical standards of the Declaration of Helsinki. The protocol was approved by the College of Medicine Research and Ethics Committee, Malawi, and the Ethical Committee of Pirkanmaa Hospital District, Finland. This trial has been registered at www.clinicaltrials.gov (identifier NCT00131235).

## RESULTS

### Enrollment and background.

Between December 1, 2003, and October 11, 2006, 1,320 women were enrolled in the study and randomized to control (436), monthly SP (441), and AZI-SP groups (443). At enrollment the intervention groups were similar except for small differences in the prevalence of microscopic malaria parasitemia and mean number of previous pregnancies (Table 1).

**Table 1:** Baseline characteristics of the participating women at enrollment, by study group

Characteristic	Control (SP twice)	Monthly SP	AZI-SP
No. enrolled women	436	441	443
Mean (SD) age (years)	25 (7)	25 (7)	25 (6)
Mean (SD) height (cm)	155.0 (5.5)	154.8 (5.4)	155.3 (5.6)*
Mean (SD) BMI (kg/m^2^)	21.7 (2.2)	21.8 (2.1)	21.9 (2.1)*
Mean (SD) gestational age at enrollment (weeks)	20.3 (3.0)	20.0 (3.2)	20.0 (3.0)
No. previous pregnancies (%)			
0	110 (25.2)	107 (24.3)	89/442 (20.1)
1	86 (19.7)	78 (17.7)	82/442 (18.6)
≥ 2	240 (55.1)	256 (58.1)	271/442 (61.3)
Proportion of HIV-positive (%)	48/396 (12.1)	64/400 (16.0)	49/398 (12.3)
Proportion of positive syphilis status (%)	18/433 (4.2)	27/435 (6.2)	21/440 (4.8)
Mean (SD) blood Hb concentration (g/L)	111 (19)	111 (17)	110 (20)
Moderate or severe anemia (Hb < 100 g/L) (%)	116 (26.6)	106 (24.0)	129 (29.1)
Severe anemia (Hb < 70 g/L) (%)	9 (2.1)	2 (0.5)	9 (2.0)
Proportion with microscopic peripheral blood malaria parasitemia (%)	49/435 (11.3)	41 (9.3)	27 (6.1)
Proportion with PCR diagnosed malaria parasitemia (%)	187/433 (43.2)	167/437 (38.2)	178/439 (40.5)
Proportion of literate participants (%)	116 (26.6)	129 (29.3)	139 (31.4)
Mean (SD) years of schooling completed	2 (3)*	2 (3)	2 (3)
Proportion of those owning any type of bed net (%)	320 (73.4)	318 (72.1)	330 (74.5)
Proportion who used bed net during previous night (%)	268 (61.5)	262 (59.4)	267 (60.3)
No. (%) twin pregnancies in this study	3 (0.7)	2 (0.5)	2 (0.5)

AZI = azithromycin; BMI = body mass index; Hb = hemoglobin; PCR = polymerase chain reaction; SP = sulfadoxine-pyrimethamine.

* Value missing for one participant.

**Figure 1.**
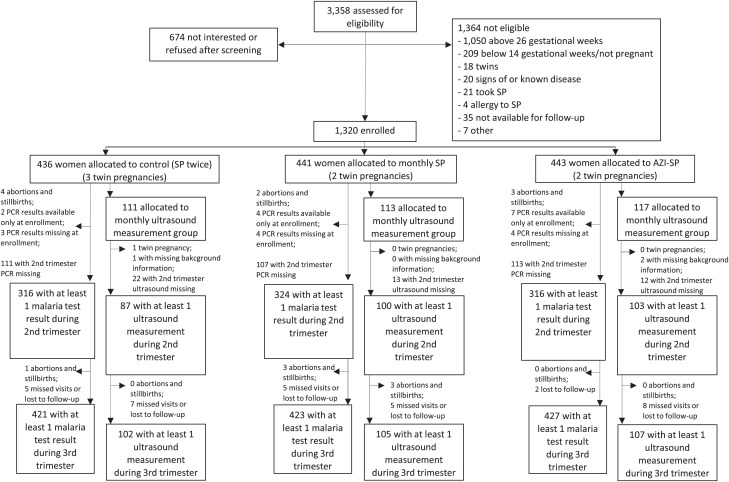
Enrollment, randomization, and follow-up. AZI-SP = intervention group with monthly sulfadoxine-pyrimethamine (SP) and two doses of azithromycin; PCR = polymerase chain reaction.

The mean (SD) number of scheduled SP treatments received, among those included in these analyses, was 2.0 (0.2) in the control group, 4.0 (0.9) in the monthly SP group, and 4.0 (0.8) in the AZI-SP group. Women in the AZI-SP group received a mean (SD) of 2.0 (0.2) azithromycin doses. A DBS sample at enrollment and at least once thereafter with all covariate data was available from 97.5% of the participants. Excluding DBS sample at enrollment, the mean (SD) number of DBS samples included in the analyses during the second and third trimesters were 1.4 (0.5) and 2.5 (0.8) samples per participant, respectively, with no difference between groups. Each participant in the monthly ultrasound follow-up group had at least one ultrasound measurement after enrollment (Figure 1). Excluding enrollment, the mean (SD) number of ultrasound measurements included in the analyses during the second and third trimesters were 1.4 (0.5) and 2.0 (0.8) measurements per participant, respectively, with no difference between groups.

### Maternal malaria parasitemia.

**Figure 2.**
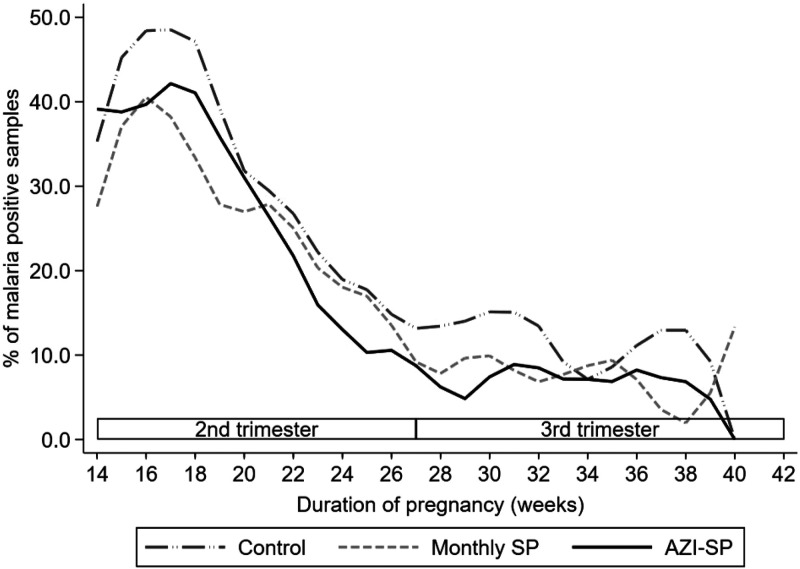
Proportion of malaria parasitemia positive samples, using the locally weighted scatterplot smoothing technique with bandwidth (0.2). AZI-SP = intervention group with monthly sulfadoxine-pyrimethamine (SP) and two doses of azithromycin.

The overall proportion (*n*/*N*) of malaria parasitemia–positive DBS samples was 40.6% (532/1,309) at enrollment, 10.5% (138/1,321) during the second trimester (excluding enrollment), and 8.8% (278/3,146) during the third trimester. There was a sharp decline in the proportion from the peak at about 16–18 gestational weeks to the beginning of the third trimester; then the decline slowed down (Figure 2). For the entire follow-up period, women in the monthly SP group had an OR (95% CI) of 0.64 (0.42–0.98; *P* = 0.040), and women in the AZI-SP group had an OR of 0.50 (0.32–0.76; *P* = 0.001) for having a positive result in malaria parasitemia test compared with the control group (Table 2). Estimated marginal risk ratios (95% CI) for the monthly SP and AZI-SP groups compared with the control were 0.75 (0.55–0.96) and 0.63 (0.45–0.81), respectively. Stratified by the duration of pregnancy, the OR for a positive malaria parasitemia test in the monthly SP group was 0.79 (0.46–1.37; *P* = 0.399) in the second trimester and 0.58 (0.37–0.92; *P* = 0.021) in the third trimester. In the AZI-SP group, the corresponding ORs were 0.47 (0.26–0.84; *P* = 0.012) and 0.51 (0.32–0.81; *P* = 0.004) in the second and third trimesters of pregnancy, respectively (Table 2). The ORs between the AZI-SP and the monthly SP groups were 0.77 (0.49–1.20; *P* = 0.248) during the entire follow-up period and were 0.59 (0.31–1.13; *P* = 0.110) and 0.87 (0.53–1.43; *P* = 0.591) during the second and third trimesters, respectively (Table 2). There were no statistically significant differences in the ORs between the second and third trimesters of pregnancy (each *P* > 0.05; details not shown). A sensitivity analysis including maternal malaria parasitemia at delivery did not change the results (Table 2).

**Table 2:** Proportion of maternal malaria parasitemia by intervention group during the whole pregnancy and the second and third trimesters of pregnancy separately

% of positive malaria parasitemia tests	Control (SP twice), *n*/*N* (%)*	Monthly SP, *n*/*N* (%)*	AZI-SP, *n/N* (%)*	Global *P* value†	Comparison between AZI-SP and control group		Comparison between AZI-SP and monthly SP group		Comparison between monthly SP and control group	
					OR† (95% CI)	*P* value†	OR† (95% CI)	*P* value†	OR† (95% CI)	*P* value†
Throughout pregnancy	177/1,447 (12.2)	126/1,489 (8.5)	108/1,497 (7.2)	0.005	0.50 (0.32–0.76)	0.001	0.77 (0.49–1.20)	0.248	0.64 (0.42–0.98)	0.040
Second trimester‡	55/424 (13.0)	48/445 (10.8)	33/441 (7.5)	0.041	0.47 (0.26–0.84)	0.012	0.59 (0.31–1.13)	0.110	0.79 (0.46–1.37)	0.399
Third trimester‡	122/1,023 (11.9)	78/1,044 (7.5)	75/1,056 (7.1)	0.008	0.51 (0.32–0.81)	0.004	0.87 (0.53–1.43)	0.591	0.58 (0.37–0.92)	0.021
Exploratory analysis: including dried blood spots taken at delivery										
Throughout pregnancy	209/1,608 (13.0)	136/1,639 (8.3)	116/1,667 (7.0)	< 0.001	0.43 (0.29–0.64)	< 0.001	0.75 (0.49 to 1.14)	0.174	0.58 (0.39 to 0.85)	0.006
Second trimester	55/424 (13.0)	48/445 (10.8)	33/441 (7.5)	0.030	0.46 (0.26–0.82)	0.008	0.60 (0.32–1.11)	0.105	0.78 (0.46–1.32)	0.351
Third trimester	154/1,184 (13.0)	88/1,194 (7.4)	83/1,226 (6.8)	< 0.001	0.42 (0.27–0.64)	< 0.001	0.82 (0.52–1.31)	0.414	0.51 (0.33–0.77)	0.002

AZI = azithromycin; OR = odds ratio; SP = sulfadoxine-pyrimethamine.

* Unadjusted proportions.

† Estimated with mixed-effects logistic regression, adjusted for polymerase chain reaction test result at enrollment, number of previous pregnancies, and duration of pregnancy at the time of malaria test.

‡ Second trimester from 14 weeks + 0 days of gestation to 26 weeks + 6 days of gestation; third trimester from 27 weeks + 0 days of gestation to the end of pregnancy.

There was no statistically significant interaction on the proportion of malaria parasitemia between the intervention group and number of previous pregnancies (*P* = 0.204), maternal HIV status (*P* = 0.319), or bed net use at enrollment (*P* = 0.990). The proportion of positive malaria parasitemia tests was lower in the AZI-SP group than in the control group in all subsamples stratified by maternal parity, HIV status, or bed net use. The OR (95% CI) of having a positive malaria parasitemia result in the AZI-SP group compared with the control was 0.24 (0.07–0.83) among HIV-positive women and 0.59 (0.37–0.94) among HIV-negative women. Corresponding ORs (95% CI) among primiparous and multiparous women were 0.38 (0.16–0.90) and 0.58 (0.35–0.95), respectively, and among those using or not using a bed net at enrolment were 0.46 (0.25–0.85) and 0.52 (0.28–0.95), respectively. The differences between the AZI-SP and the control group were statistically significant among primiparous (*P* = 0.027, global *P* = 0.043) and those who used bed net (*P* = 0.012, global *P* = 0.037) (Table 3). The associations were in the same direction but smaller between the monthly SP and the control groups, with statistically significant differences among primiparous women (OR = 0.44, 95% CI = 0.20–0.99; *P* = 0.046) (Table 3).

**Table 3:** Proportion of maternal malaria parasitemia by intervention group throughout pregnancy, stratified by number of previous pregnancies, HIV status, and use of bed net during previous night before enrollment

Interaction by	Stratified by	Control (SP twice), *n*/*N* (%)*	Monthly SP, *n*/*N* (%)*	AZI-SP, *n*/*N* (%)*	Global *P* value†	Comparison between AZI-SP and control group		Comparison between AZI-SP and monthly SP group		Comparison between monthly SP and control group	
						OR† (95% CI)	*P* value†	OR† (95% CI)	*P* value†	OR† (95% CI)	*P* value†
Maternal HIV	HIV−	128/1,155 (11.1)	90/1,119 (8.0)	84/1,181 (7.1)	0.071	0.59 (0.37–0.94)	0.025	0.83 (0.51–1.34)	0.449	0.71 (0.45–1.12)	0.143
	HIV+	34/148 (23.0)	29/217 (13.4)	13/169 (7.7)	0.077	0.24 (0.07–0.83)	0.024	0.47 (0.14–1.56)	0.220	0.50 (0.17–1.49)	0.214
No. of previous pregnancies	Multiparous	100/1,902 (9.2)	81/1,150 (7.0)	74/1,209 (6.1)	0.098	0.58 (0.35–0.95)	0.031	0.76 (0.46–1.27)	0.290	0.76 (0.46–1.24)	0.276
	Primiparous	77/355 (21.7)	45/339 (13.3)	34/288 (11.8)	0.043	0.38 (0.16–0.90)	0.027	0.87 (0.35–2.12)	0.753	0.44 (0.20–0.99)	0.046
Bed net use during previous night	Used bed net	99/903 (11.0)	64/882 (7.3)	56/914 (6.1)	0.037	0.46 (0.25–0.85)	0.012	0.74 (0.40–1.39)	0.348	0.63 (0.38–1.12)	0.113
	Did not use bed net	78/544 (14.3)	62/607 (10.2)	52/583 (8.9)	0.096	0.52 (0.28–0.95)	0.034	0.79 (0.42–1.48)	0.466	0.65 (0.36–1.19)	0.161

AZI = azithromycin; OR = odds ratio; SP = sulfadoxine-pyrimethamine.

* Unadjusted proportions.

† Estimated with mixed-effects logistic regression, adjusted for malaria parasitemia status (polymerase chain reaction) at enrollment, number of previous pregnancies (except when stratified by number of previous pregnancies), and duration of pregnancy at the time of malaria test.

### Fetal growth.

**Figure 3.**
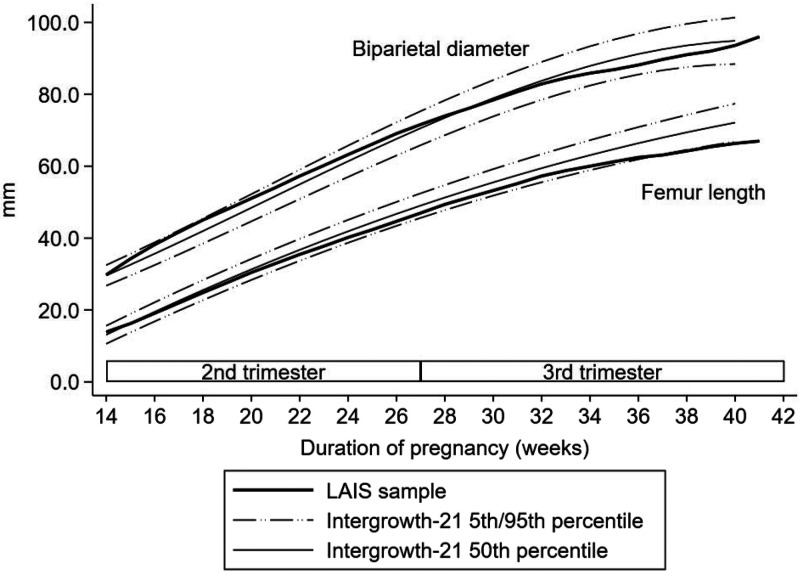
Mean fetal biparietal diameter (mm) and femur length (mm) compared with INTERGROWTH-21st standards, using the locally weighted scatterplot smoothing technique with bandwidth (0.2). LAIS = Lungwena Antenatal Intervention Study.

The mean (SD) biparietal diameter of the fetuses was 41.8 mm (7.4) at enrollment, 77.1 mm (4.6) at 28–32 weeks of pregnancy, and 90.7 mm (4.3) at 37–38 weeks of pregnancy. Corresponding values for femur length were 22.5 mm (6.1), 52.0 mm (4.0), and 63.3 mm (4.3), respectively. The mean biparietal diameter was close to the 95th percentile of INTERGROWTH-21st standards at the beginning of the second trimester of pregnancy but fell below the 50th percentile toward the end of pregnancy. The mean femur length was close to the 50th percentile of INTERGROWTH-21st standard at the beginning of the second trimester but fell to the 5th percentile toward the end of pregnancy (Figure 3).

The estimated mean (standard error [SE]) change in biparietal diameter per 4 weeks during the second trimester of pregnancy was 12.4 mm (0.3), 12.0 mm (0.3), and 11.9 mm (0.3) in the control, monthly SP, and AZI-SP groups, respectively. Corresponding changes in biparietal diameter during the third trimester of pregnancy were 7.1 mm (0.3), 7.0 mm (0.2), and 6.9 mm (0.2). There were no statistically significant differences between the intervention groups during either of the trimesters (each *P* > 0.05) (Table 4).

**Table 4:** Change in fetal biparietal diameter (mm) and femur length (mm) per 4 weeks by intervention group during the second and third trimesters of pregnancy

Outcome	Trimester*	Control (SP twice), mean (SE)†	Monthly SP, mean (SE)†	AZI-SP, mean (SE)†	Global *P* value†	Comparison between AZI-SP and control group		Comparison between AZI-SP and monthly SP group		Comparison between monthly SP and control group	
						Difference in means† (95% CI)	*P* value†	Difference in means† (95% CI)	*P* value†	Difference in means† (95% CI)	*P* value†
Biparietal diameter (mm/4 weeks)	Second	12.4 (0.3)	12.0 (0.3)	11.9 (0.3)	0.521	−0.5 (−1.4 to 0.4)	0.262	−0.2 (−1.0 to 0.7)	0.708	−0.4 (−1.2 to 0.5)	0.428
	Third	7.1 (0.3)	7.0 (0.2)	6.9 (0.2)	0.884	−0.2 (−0.8 to 0.5)	0.622	−0.1 (−0.7 to 0.6)	0.775	−0.1 (−0.8 to 0.6)	0.830
Femur length (mm/4 weeks)	Second	9.4 (0.4)	9.9 (0.3)	9.1 (0.3)	0.149	−0.4 (−1.3 to −0.6)	0.446	−0.8 (−1.7 to 0.0)	0.053	0.5 (−0.4 to 1.4)	0.275
	Third	6.7 (0.3)	6.0 (0.2)	6.1 (0.2)	0.109	−0.6 (−1.3 to 0.1)	0.105	0.1 (−0.5 to 0.8)	0.677	−0.7 (−1.5 to −0.0)	0.045
Exploratory analysis: including measurements taken before 38 weeks of gestation											
Biparietal diameter (mm/4 weeks)	Third	7.2 (0.3)	7.5 (0.3)	7.4 (0.3)	0.788	0.1 (−0.6 to 0.9)	0.687	−0.1 (−0.8 to 0.6)	0.767	0.3 (−0.5 to 1.0)	0.491
Femur length (mm/4 weeks)	Second	7.1 (0.3)	6.6 (0.3)	6.3 (0.3)	0.129	−0.8 (−1.5 to −0.0)	0.046	−0.3 (−1.0 to 0.5)	0.503	−0.5 (−1.3 to 0.3)	0.186

AZI = azithromycin; OR = odds ratio; SP = sulfadoxine-pyrimethamine; SE = standard error.

* Second trimester from 14 weeks + 0 days of gestation to 26 weeks + 6 days of gestation; third trimester from 27 weeks + 0 days of gestation to the end of pregnancy.

† Estimated with mixed-effects linear regression, adjusted for ultrasound measurement at enrollment, number of previous pregnancies, malaria parasitemia status (polymerasce chain reaction) at enrollment, maternal body mass index at enrollment, and duration of pregnancy at time of ultrasound measurement.

Estimated mean (SE) change in femur length per 4 weeks during the second trimester was 9.4 mm (0.4), 9.9 mm (0.3), and 9.1 mm (0.3) mm in the control, monthly SP, and AZI-SP groups, respectively. Corresponding changes in femur length during the third trimester of pregnancy were 6.7 mm (0.3), 6.0 mm (0.2), and 6.1 mm (0.2). There were no statistically significant differences between the intervention groups during either of the trimesters (each *P* > 0.05) (Table 4).

A sensitivity analysis excluding biparietal diameter and femur length values measured at or after 38 weeks of gestation did not change the results (Table 4).

## DISCUSSION

The aim of this study was to determine the impact of antenatal monthly SP with or without two doses of azithromycin on maternal PCR-diagnosed malaria parasitemia and fetal biparietal diameter and femur length growth velocity in the second and third trimesters of pregnancy compared with two doses of SP. In a sample of 1,320 rural Malawian women, the odds of malaria parasitemia during the second and third trimesters separately or combined were up to 50% lower among participants treated with monthly SP or AZI-SP compared with those in the control group. There were no statistically significant interactions between malaria parasitemia and maternal HIV status, parity, or bed net use. However, the odds of malaria parasitemia were 50–76% lower among HIV-positive and primiparous women in the monthly SP and AZI-SP groups compared with those in the control group, with statistically significant differences among primiparous women in both intervention groups compared with the control. There were no differences between the groups in the growth velocity of biparietal diameter or femur length.

The strengths of this trial include random group allocation, broad inclusion criteria, a large sample size, comprehensive follow-up, and blinding of the outcome assessors. Internal validity could have been compromised by variation in the timing of DBS collection in terms of 1) gestational weeks and ultrasound measurements during pregnancy, 2) the small baseline differences in the prevalence of maternal malaria parasitemia and number of previous pregnancies between the intervention groups, and 3) that the study was not powered to detect small differences between the monthly SP and the AZI-SP groups or find interactions for the outcomes. However, we used all available malaria parasitemia and ultrasound measurements as outcomes, regardless of the timing, using mixed effect models that allowed this. All the models adjusted for variables with imbalance at baseline. Thus, we believe these factors did not bias our conclusions, and we consider the findings indicative of a causal association between the antenatal monthly SP and AZI-SP interventions and reduced maternal malaria parasitemia prevalence during pregnancy. The impact may have been larger among primiparous or HIV-positive women than among multiparous or HIV-negative women, but the study was not sufficiently powered to make firm conclusions from the subgroup analyses. In contrast, the findings do not provide support for a hypothesis that either of the interventions would affect biparietal diameter or femur length growth velocity.

Our results are in line with other studies that have shown a positive impact of monthly SP on maternal malaria during pregnancy compared with the two-dose SP regimen.^11^^,^^23^^,^^24^ We have not found studies that looked at the impact of monthly SP during the second and third trimesters separately, but there is no biological reason to believe the results would differ from our findings. Although azithromycin has some antimalarial activity,^25^ previous results about the impact of azithromycin on malaria are mixed, with some studies indicating a reduction in malaria^26^^–^^28^ and some studies suggesting no impact.^25^^,^^29^^,^^30^ Our results suggest that there is no major impact of azithromycin on maternal malaria parasitemia when combined with IPTp with monthly SP in Malawi.

Although maternal malaria has been shown to have a negative impact on fetal and newborn head size and length,^31^^,^^32^ we did not see differences in biparietal diameter or femur length gain velocity between any of the intervention groups, despite the lower odds of malaria parasitemia among women treated with monthly SP. We have previously shown that women in the AZI-SP group had pregnancies that were on average 3 days longer and that their infants were on average 6 mm longer and had a head circumference that was on average 4 mm larger than infants born to women in the control group.^12^^,^^13^ Given a mean fetal length gain of approximately 1 cm per week toward the end of pregnancy,^33^ the 3-day difference in the duration of pregnancy translates to approximately 5 mm difference in length at birth. These findings, combined with those from the current analysis, suggest that IPTp with AZI-SP, starting in the second trimester, increases mean infant length at 1 month of age by extending the mean duration of pregnancy but not by increasing the velocity of length gain.

Because we did not measure fetal abdominal circumference in this study, we could not use ultrasound assessment to calculate fetal weight gain during pregnancy in the way we assessed gains in length. However, we have previously reported that infants born to mothers in the AZI-SP group had about 40% lower incidence of LBW and 140 g higher mean birth weight than infants born to mothers in the control group.^12^^,^^13^ Statistical models suggested that approximately one-third of the difference in birth weight was due to a difference in the duration of pregnancy and two-thirds to a difference in weight gain velocity.^13^ The differential impact of the IPTp interventions on length and weight gain velocity may be related to fetal growth kinetics (i.e., that fetal length and head circumference peak in the second trimester of pregnancy but weight gain only in the third trimester).^34^^,^^35^ If IPTp with AZI-SP or monthly SP was started already in the first trimester, a bigger impact might be possible also on fetal gains in length and head circumference.

Earlier results from this trial have shown that antenatal AZI-SP treatment reduced the incidence of preterm birth, LBW, and incidence of stunting during the first 5 years of a child’s life compared with two doses of SP.^12^^,^^14^ Monthly SP alone also increased mean birth weight, but the increase was bigger in the AZI-SP group.^13^ Because there were no differences between the monthly SP and AZI-SP groups in the odds of PCR-diagnosed malaria parasitemia during pregnancy, the results suggest that the additional impact of azithromycin on the duration of pregnancy and birth size is likely not mediated through an antimalarial mechanism. Alternative mechanisms include at least azithromycin’s antibacterial and anti-inflammatory activity,^36^ which may block pathways leading to fetal growth restriction and preterm birth.^6^^,^^37^ Along these lines, authors from a Papua-New Guinean trial concluded that IPTp with AZI-SP would improve birth outcomes through its impact on inflammation and placental angiogenesis.^38^ The antibacterial mechanism seems especially feasible given the broad spectrum of azithromycin, but the same antibacterial pathway may be important also for the narrower-spectrum SP. This is suggested by data from a Kenyan trial, in which IPTp with a new and potent antimalarial, dihydroartemisinin-piperaquine, rather than SP was associated with a much better maternal malaria control during pregnancy but a markedly lower mean birth weight.^39^

After the implementation of our study 15–20 years ago, the recommendation for IPTp has changed from two doses to monthly dosing with SP.^10^ Additionally, HIV-positive women nowadays receive antiretroviral therapy, as opposed to the single-dose nevirapine regimen used for PMTCT at the time of our study. Eligible women also receive co-trimoxazole prophylaxis, in which case they should not receive IPTp or malaria treatment containing SP.^40^^,^^41^ Also, the possible SP resistance of *P. falciparum* and spreading antibiotic resistance should be considered.^42^^–^^46^ Although the results from our sample suggest that azithromycin combined with monthly SP would provide benefits in birth outcomes compared with the monthly SP alone, the policy changes and increased SP and antibiotic resistance might modify its impact on one or more outcomes.

In conclusion, results from this study support the hypothesis that monthly SP with or without two doses of azithromycin reduced maternal malaria parasitemia during pregnancy in Malawi. In contrast, the study findings did not provide evidence that the same interventions would affect growth velocity of fetal biparietal diameter and femur length. When combined with earlier results on birth size, the findings suggest that the AZI-SP intervention increased mean infant length at 1 month of age mainly by extending the duration of pregnancy and increased birth weight also by increasing the speed of fetal weight gain. The addition of azithromycin to monthly SP did not greatly improve the antimalarial effect of the IPTp regimen but further improved birth outcomes through other mechanisms, probably through antibacterial and anti-inflammatory pathways.
